# How destination brand experience influences tourist citizenship behavior: Testing mediation of brand relationship quality and moderation effects on commitment

**DOI:** 10.3389/fpsyg.2023.1080457

**Published:** 2023-03-09

**Authors:** Jinwen Tang, Jingna Wang, Min Zhang, Weizhao Huang

**Affiliations:** ^1^College of Management, Guangdong Polytechnic Normal University, Guangzhou, China; ^2^International College, Krirk University, Bangkok, Thailand; ^3^College of Tourism and Service Management, Nankai University, Tianjin, China; ^4^China University of Petroleum (East China), Qingdao, China; ^5^Guangdong Literature and Art Vocational College, Guangzhou, China

**Keywords:** brand experience, tourist citizenship behavior, brand relationship quality, commitment, tourism destination management

## Abstract

This study examines the potential predictors of tourist citizenship behavior based on the Stimulus–Organism–Response framework. The studies were conducted in China. Data were collected *via* questionnaire surveys. Structural equation path modeling and mediation as well as moderation role were used for data analyses. This model was used to test the hypotheses using a sample of 325 individuals with tourism experience in Guangzhou city. The results reveal that tourism destination brand experience and brand relationship quality significantly affect tourist citizenship behavior. Furthermore, the results show that brand relationship quality significantly mediates the relationship between tourism destination brand experience and tourist citizenship behavior and demonstrate that commitment plays a significant moderating role between brand relationship quality and tourist citizenship behavior. This study clearly shows the relationship between tourism destination brand experience, brand relationship quality, and tourist citizenship behavior. Thus, this study contributes to existing tourism studies by identifying gaps and proposing a holistic view to understand tourist citizenship behavior in the tourism industry.

## 1. Introduction

The concepts of the customer as the company’s “good soldier” and customer citizenship behavior (CCB) have been studied for more than a decade after Groth (2005) suggested CCB as voluntary and autonomous behaviors in the service industry, laying the groundwork for the field, in which behaviors are not necessary for the successful production and/or delivery of services but help service organizations in general. Citizenship behaviors involve additional role behaviors, including behaviors toward other customers, employees, and organizations. In previous research, citizenship behaviors were used in the hospitality industry. Yi et al. (2011) showed that CCB directly and positively impacts employee performance and commitment.

Although citizenship behaviors play a critical role in service delivery (Morrison, 1996), research on citizenship behaviors has almost exclusively focused on employees rather than tourism. This gap in the literature is surprising, given that organizations increasingly conceptualize tourism as “partial customers” (Bowen et al., 2000) and view effective management of tourism as a strategic advantage (Lengnick-Hall, 1996).

Moreover, tourism and travel experiences “often go far beyond temporary stays and local consumption” (Mostafanezhad and Norum, 2018). This shows the importance of brand relationship quality in the tourism industry. Fournier (1998) believed that the quality of the brand relationship is an important factor in strategic brand management. This includes tourism experience and other positive behaviors derived from citizenship, such as commitment sharing among family, friends, strangers, or the destination itself (Mccable and Stokoe, 2010).

Previous sustainability studies have applied limited theories to understand the psychological mechanism of tourism destination brand experience and brand relationship quality. Smith (2002) also pointed out that existing sustainability research that applies the aforementioned theories still provides a limited explanation of brand experience in the marketing practice. The researchers then developers’ attitudes and behaviors (Thrash and Elliot, 2003).

The present study seeks to address this gap in the literature. The objectives of this paper are fourfold. The research objective of this study is to examine the effect of brand relationship quality on tourism destination brand experience and tourist citizenship behavior toward Guangzhou city setting by answering the following research questions: (a) Does tourism destination brand experience significantly influence brand relationship quality? (b) Does brand relationship quality significantly influence tourist citizenship behavior? (c) Does brand relationship quality fully or partially mediate the relationship between tourism destination brand experience and tourist citizenship behavior? More specifically, we focus on the moderating role of a citizen’s commitment to the tourism provider. Tourists with a high level of commitment maintain a relationship with a tourism provider because of the high economic switching costs and constraints rather than because of an emotional attachment to the destination (Bansal et al., 2004). Therefore, (d) Does commitment fully or partially moderate the relationship between brand relationship quality and citizenship behavior?

The main contribution of this research is to show that this study expand knowledge about tourist citizenship behavior toward tourism in Guangzhou city and emphasize the role and importance of brand relationship quality, which links the relationship between tourism destination brand experience and tourist citizenship behavior. In this regard, the study is a response to several recent calls in the literature to examine the consequences of destination brand experience for the tourist citizenship behavior implemented in tourist industry. The results of this study provide guidelines for tourists who want to visit Guangzhou city by offering information about how tourism destination brand experience affects tourist citizenship behavior through intrinsic motivation, such as brand relationship quality.

## 2. Literature review

### 2.1. S-O-R framework

The current study proposes the integration of the Stimulus-Organism-Response (S-O-R) framework (Mehrabian and Russell, 1974) to enhance the knowledge of brand relationship quality in the tourism industry.

In the tourism literature, numerous researchers have applied and extended the S-O-R framework to the restaurant context (Heung and Gu, 2012). As suggested by Turley and Milliman (2000), studying the S-O-R framework and the effects of external experiential stimuli within the brand experience is necessary because the effects of stimuli result in different responses according to the context. According to Fournier (1998), brand relationship quality should be considered an important factor within the brand experience literature because brand experience significantly affects the sensory experience, affective experiences, and behavioral and intellectual experiences of tourists. Moreover, previous studies have also recommended the potentially significant mediating role of brand relationship quality between brand experience (Bowden, 2009) and citizenship behavior (Bowen, 2001). However, only anecdotal evidence for the relationship between tourism destination brand experience (stimulus), brand relationship quality (organism), and tourist citizenship behavior (response) is available.

Thus, the current study adopted brand relationship quality as an influencer (mediator). Commitment plays the moderating role to explain tourism in Guangzhou city in the relationship between tourism destination brand experience and tourist citizenship behavior by integrating the S-O-R framework.

The S-O-R framework indicates that cues (stimulus) perceived from the environment can trigger a person’s internal evaluation state (organism), which in turn produces positive or negative behavior (responses) to the stimulus (Mehrabian and Russell, 1974). This means that the S-O-R framework aims to describe individual behavior by creating a stimulus that produces cognitive and emotional states, which in turn lead to a response. Stimuli are influencing factors in the external environment, which can affect the psychological and cognitive state of the organism (Lin and Lo, 2016). After a series of activities in psychological or cognitive areas, the body will make internal or external behavioral responses to external stimuli. Internal responses are expressed as individual attitudes, and external responses are expressed as individual-specific behaviors (Lorenzoromero, 2016). This framework suggests that the organism (brand relationship quality) provokes a response (tourist citizenship behavior) based on stimulus from external experiential cues (tourism destination brand experience).

The S-O-R framework is a meta-theory for analyzing the behavior of users that has been more extensively used in the fields of information about science and information management than in the tourism industry. As a well-known framework, previous studies have used the S-O-R framework to explain consumer loyalty, customers’ behaviors, customer experience, purchase behavior, etc. Based on the S-O-R model, Wu and Li (2018) developed a comprehensive model to explore the impact of six marketing mix components (stimulus) on consumer behaviors (response) through consumer value (organism) in social commerce. In particular, Herrando et al. (2019) analyzed how hedonic and utilitarian stimuli affect users’ traffic experience, which positively affects emotional and behavioral loyalty, combined with the moderating role of the cultural context in social commerce. Fournier (1998) believed that the quality of brand relationships is an important factor in strategic brand management. She developed a scale to measure the quality of brand relationships. Morgan-Thomas and Veloutsou (2011) found that a positive brand experience leads to repeated interactions with the same place as well as positive word of mouth about the place. Recently, a new concept of brand experience has emerged, which provides a more complete assessment based on the sensory, affective, intellectual, and behavioral dimensions of a brand (Brakus et al., 2009). Thus, tourism destination brand experience, including sensory, affective, intellectual, and behavioral can be considered a stimulus in the S-O-R framework in the tourism industry.

In these studies, the S-O-R framework, as an important analytical framework for explaining human behavioral processes, is used to predict brands’ cognitive judgments and customers’ behaviors. Its use in the tourism industry has been limited and only anecdotal evidence is available on the relationship between the tourism destination brand experience (stimulus), brand relationship quality (organism), and tourist citizenship behavior (response). The current study applies the S-O-R framework to the tourism industry to examine the effect of tourism destination brand experience on tourist citizenship behavior combined with the mediating role of brand relationship quality.

### 2.2. Brand relationship quality

Brand relationship quality (BRQ) has been described as a customer-based measure of the strength and depth of personal brand relationships (Smit et al., 2007). This concept has attracted increasing academic interest in recent years. Brand researchers have traditionally assessed how consumers perceive and value brands by examining brand attitudes, brand evaluations, or perceived brand quality (Keller, 2003). Some would say that successful branding can increase gross profit by 50% (Blumenthal, 1995). More recently, researchers have pointed out that consumers differ in their brand perceptions and relationships with brands (Muniz and O'Guinn, 2001). For example, consumers often do not distinguish between brands and brand manufacturers or representatives. In addition, marketers often try to induce consumers to see their brand as a way of life through the use of anthropomorphism, animism, and the use of human characters and other attributes (Moon, 2000).

Literature has examined different aspects of brands (e.g., brand equity, brand personality, brand image, brand loyalty), but little has been done on the quality of the relationship between tourists and brands. Customers who project themselves onto a brand show a strong attachment to the same brand (Yu et al., 2022). Blackston (1993) put forward the concept of the reciprocal relationship between brands and consumers, arguing that consumers’ perception of brand-to-consumer attitudes should be included in the study of brand image. The brand thus becomes an active partner for consumers based on the brand relationship quality. Therefore, brand relationship quality can be viewed as the transmission process that links the stimulus (e.g., tourism destination brand experience) and behavior-related activities (e.g., tourist citizenship behavior).

### 2.3. Stimulus–organism: The relationship between tourism destination brand experience and brand relationship quality

Brand experience refers to a customer’s feelings, perceptions, and behavioral responses motivated by a brand’s identity, communication, and environment (Brakus et al., 2009). Brakus et al. (2009) divided brand experience into four elements: sensory, affective, intellectual, and behavioral experience. Sensory brand experience refers to how the brand provides the customer with sensory experiences linked to the five human senses of vision, hearing, smell, taste, and touch. For example, when a tourist travels to a new tourist destination, they may first experience their environment through the ear, eye, and nose. Affective brand experience refers to the emotional experience a brand evokes. For example, a tourist may travel to a famous tourist destination. They may have positive feelings about the warm welcome of the locals (i.e., happiness, comfort, enjoyment) or negative feelings about the indifferent reactions of the locals (i.e., anxiety, sadness, depression). Intellectual brand experience mainly refers to the cognitive experience provided by the brand. For example, tourists are curious about the tourist destination’s culture, history, landscape, etc. (Tang et al., 2019). behavioral brand experience refers to the experience that a brand provides through actions and behaviors. For example, tourists are concerned with the urban public facilities and tourist environment of the destination. Brakus et al. (2009) tested empirical research models and found that measures of brand experience differ from brand attitudes, engagement, personality, and attachment. They also found that brand experience significantly affects brand personality, satisfaction, and loyalty.

Fournier’s (1998) conceptualisation of the brand relationship quality framework is probably the most frequently cited work in the area of brand relationship quality. Fournier found that consumers do not buy brands just because the brands work well. They engage in relationships with a group of brands to benefit from the meaning these brands add to their lives. Some of these meanings are functional and practical; others are psychological and emotional. This process is purposeful, self-centered, and means a lot to those involved. Important factors in maintaining relationships are emotional and socio-emotional attachment (love/passion and self-connection), behavioral connection (interdependence and commitment), and supportive cognitive beliefs (intimacy and brand partner quality). These factors combine to create strength and durability over time.

Brand relationship quality consists of the following aspects, according to research: self-concept connection, love, personal commitment, intimacy, and passionate attachment.

In other words, tourism destination brand experiences include four factors: sensory, affective, intellectual, and behavioral experiences, may help shape brand relationship quality, which is intrinsic motivation. As a result, tourists with sensory, affective, intellectual, and behavioral experiences in destination brand experience may be more concerned with brand relationship quality than in forming attitudes and behavioral responses. Andreini et al. (2018) criticized boundaries in the brand experience literature; however, there is limited research on the impact of brand experience on intrinsic motivation. Although previous research has suggested possible future studies and provided anecdotal evidence that suggested brand experience can positively influence brand relationship quality (Zarantenello and Schmitt, 2000), there is limited empirical research available for the accurate tourism destination brand experience factors influence brand relationship quality. Therefore, this study uses an S-O-R framework to improve our understanding of four factors about brand experience in the tourism destination which influence brand relationship quality. Therefore, we propose the following hypothesis.

*H1a*: Sensory experience positively influences brand relationship quality.

*H1b*: Affective experience positively influences brand relationship quality.

*H1c*: Intellectual experience positively influences brand relationship quality.

*H1d*: behavioral experience positively influences brand relationship quality.

### 2.4. Organism–responses: The relationship between brand relationship quality and tourist citizenship behavior

Organizational citizenship behavior is spontaneous and beneficial to the organization (Organ, 1988). These positive behaviors are critical because they act as a social lubricant that can smooth out many unforeseen contingencies (Smith et al., 1983) while enhancing the social and psychological environment that supports task performance (Organ, 1997).

Current research focuses on the civic behavior of tour group members (tourists rather than employees). What happens with organizational citizenship behavior and tourists’ citizenship behavior is similar. As customers are active participants in the service production process, there is a social context to developing team dynamics in tour groups. Group dynamics may influence both types of citizen behavior.

Although research on tourists’ citizenship behavior is limited, several studies reveal this phenomenon through studies of customers’ citizenship behavior and brand relationship quality interactions. Customers’ citizenship behavior encourages them to voluntarily offer products, advise other customers, and provide feedback to the brand. According to the social exchange theory, in the case of the brand relationship quality in the tourist destination, the tourists’ satisfaction, degree of commitment, trust, attachment, and emotional intimacy with the brand will be higher (Cheng et al., 2016). Customers also tend to take positive actions, such as volunteering to help the organization (i.e., organization-directed citizen behavior) and other tourists in the organization (i.e., customer-directed citizen behavior). Studies have shown that the quality of brand relationship affects tourists’ behavioral responses, such as greater revisit intention, more desire to travel to the city, willingness to share personal information with the organization, and tendency to provide positive word of mouth and brand support (Shen, 2013). Therefore, the brand relationship quality influences on tourism citizenship behavior, tourism citizenship has two factors on customer-directed citizen behavior and organization-directed behavior.

However, empirical research on the relationship between brand relationship quality and tourist citizenship behavior is limited. Therefore, we propose the following hypothesis.

*H2a*: Brand relationship quality has a positive impact on customer-directed citizen behaviour.

*H2b*: Brand relationship quality has a positive impact on organisation-directed citizen behaviour.

### 2.5. S-O-R: The mediating role of brand relationship quality

This study primarily assesses whether tourism destination brand experience produces brand relationship quality that affects tourists’ citizenship behavior. The idea is largely based on research gaps in the brand experience and sustainability literature. Andreini et al. (2018) pointed out some limitations of brand experience research. One such limitation is that most consequences of brand experience are related to cognitive/emotional brand-related variables, such as brand awareness and brand satisfaction, rather than brand relationship quality or customer behavior (i.e., tourist citizenship behavior). However, research on the impact of brand experience as well brand-limited research on the effects of brand relationship quality variables has been conducted.

Another limitation of the tourism destination brand experience literature is that only a few studies have applied purposeful theory to investigate tourism destination brand experience (Andreini et al., 2018). To bridge this gap, the current study initially adopted the S-O-R framework (Mehrabian and Russell, 1974), which has been widely adopted to expand the understanding of tourist journeys in tourism (Choi and Kandampully, 2019). Given the anecdotal evidence on the relationship between tourism destination brand experience, brand relationship quality, and tourist citizenship behavior, this study applied the S-O-R framework to explain the psychological mechanisms of these variables. Therefore, this study built on this framework by measuring the order effect of tourism destination brand experience (stimulus) on tourist citizenship behavior (response) through the mediating role of brand relationship quality (organism) to explain the relationship between these variables in the area of psychological mechanism.

According to social communication theory, customers feel obligated to return the favor when they benefit from another person or organization. When tourists want to gain a good sensory, affective, intellectual, and thinking experience in a tourism destination, they often get it first through organizations and individuals familiar with the tourism destination, thereby forming a brand relationship layer. In this process, the quality of the brand relationship acts as a mediating variable that mediates the tourist destination brand experience include four factors on sensory experience affective experience, intellectual experience and behavioral experience and tourist citizenship behavior on customer-directed citizen behaviors and organization-directed citizen behavior. Therefore, we propose the following hypothesis.

*H3a*: Brand relationship quality mediates the relationship between sensory experience and customer-directed citizen behaviours.

*H3b*: Brand relationship quality mediates the relationship between affective experience and customer-directed citizen behaviours.

*H3c*: Brand relationship quality mediates the relationship between intellectual experience and customer-directed citizen behaviours.

*H3d*: Brand relationship quality mediates the relationship between behavioural experience and customer-directed citizen behaviours.

*H3e*: Brand relationship quality mediates the relationship between sensory experience and organisation-directed citizen behaviours.

*H3f*: Brand relationship quality mediates the relationship between affective experience and organisation-directed citizen behaviours.

*H3g*: Brand relationship quality mediates the relationship between intellectual experience and organisation-directed citizen behaviours.

*H3h*: Brand relationship quality mediates the relationship between behavioural experience and organisation-directed citizen behaviours.

### 2.6. S-O-R: The moderating role of commitment

While commitment has been conceptualized as a multidimensional structure (Meyer and Allen, 1991), this study focuses on the affective dimension of this structure, as this dimension is a particularly strong predictor of citizen behavior (Jones et al., 2010). It argues that in addition to the entire tourism industry, providers or the followers of people related to the brand community and other tourists can also be important targets for tourists’ commitment since interactions between tourists are often an integral part of the service experience (Verhoef et al., 2009). In many service environments, especially in the tourism industry, tourists often communicate in the presence of other tourists (Thakor et al., 2008). While previous research has focused on the influence of customers interactions between strangers, customers can also develop intimacy by repeatedly sharing consumer experiences (Harris and Baron, 2004).

Jones and Taylor (2012) refer to close and committed relationships between individuals in a tourism network as high “relational social capital” and found a positive association between this form of social capital and a tourist’s attitudinal loyalty to the brand provider. Thus, the commitment to a person (e.g., a fellow customer) within the “service delivery network” can be affectively transferred to the entire brand destination city because the brand destination city is part of the social network to which the person is connected (Hansen et al., 2003).

Previous research has provided evidence that commitment to one goal within an organization can be transferred to other goals (Hansen et al., 2003). For example, Hansen et al. (2003) found that emotional commitment to providers has a positive carryover effect on the commitment to the organization as a whole. Similarly, Jones et al. (2010) found that commitment to a provider as a friend was positively related to commitment to a provider as an economic exchange partner and the organization as a whole.

**Figure 1:**
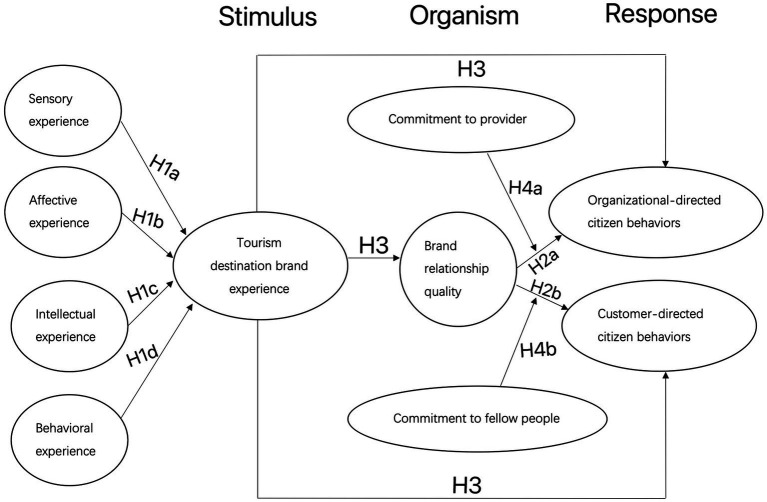
Shows the study’s proposed model that is based on the literature review.

As revealed by prior research, commitment to providers positively affects a customer’s citizen behavior (Yi and Gong, 2006). Consequently, in addition to the direct effects, the commitment can also be expected to increase organization-directed citizen behavior and customer-directed citizen behavior *via* an indirect pathway, i.e., through increased levels of commitment to the organization (Figure 1). Based on the above discussion, we propose the following hypothesis:

*H4a*: Commitment to the provider moderates the relationship between brand relationship quality and organisational-directed citizen behaviours.

*H4b*: Commitment to fellow people moderates the relationship between brand relationship quality and customer-directed citizen behaviours.

## 3. Methods

### 3.1. Data collection

To test the proposed hypothesis, we implemented a quantitative research design that employed an online survey using structured questionnaires and non-probabilistic convenience samples. A total of 500 participants who had tourism experience in Guangzhou city were randomly recruited from the online survey platform WenJuanXing, which provides functions equivalent to Amazon Mechanical Turk. Individuals can only participate in one survey, which takes approximately 10 min to complete. The data collection period for this study was from 8 March to 8 April 2022. Respondents who have read the survey’s purpose and description can participate in the survey after signing the consent form. The authors asked a preliminary question: Do you have experience in tourism in Guangzhou city? If “YES,” please complete all the remaining parts of this questionnaire. If “NO,” you do not have to proceed. The screening question was used to target customers with tourism experience in Guangzhou. The respondents also needed to provide demographic information related to gender, age, individual monthly income, occupation, and education level. Next, the questionnaire asked about tourist citizenship behavior in Guangzhou city and ended with questions about commitment to Guangzhou city.

After collecting the data, we examined the results of the questions and determined whether individual participants completed the screening process when answering the questions. Therefore, participants who did not complete the questionnaire or did not correctly answer the attention-check questions were excluded from the study. In total, valid responses were collected from 325 respondents. Table 1 shows the details of the demographic characteristics of the participants.

**Table 1:** Sociodemographic analysis of the sample (*n* = 325).

		Frequency	Percent	Valid Percent	Cumulative Percent
Gender	Male	158	48.6	48.6	48.6
	Female	167	51.4	51.4	100.0
	Total	325	100.0	100.0	
Age	18–24y	69	21.2	21.2	21.2
	15–34y	156	48.0	48.0	69.2
	35–50y	80	24.6	24.6	93.8
	Over 50y	20	6.2	6.2	100.0
	Total	325	100.0	100.0	
Monthly Individual Income (Yuan)	Under ¥5,000	40	12.3	12.3	12.3
	¥5,000–¥9,999	97	29.8	29.8	42.2
	¥10,000–¥14,999	99	30.5	30.5	72.6
	¥15,000–¥19,999	61	18.8	18.8	91.4
	Over ¥20,000	28	8.6	8.6	100.0
	Total	325	100.0	100.0	
Occupation	Company employee	112	34.5	34.5	34.5
	Business owner	52	16.0	16.0	50.5
	Professional worker	32	9.8	9.8	60.3
	Student	93	28.6	28.6	88.9
	Housewife	13	4.0	4.0	92.9
	Other	23	7.1	7.1	100.0
	Total	325	100.0	100.0	
Education	High school diploma	117	36.0	36.0	36.0
	Bachelor’s degree	142	43.7	43.7	79.7
	Graduate degree	66	20.3	20.3	100.0
	Total	325	100.0	100.0	

### 3.2. Measurement

All constructs were measured using a seven-point Likert-type scale (1 = strongly disagree to 7 = strongly agree). All the items were taken from previous literature to ensure the content validity of the structure. Tourist citizenship behavior was on the work of Bartikowski and Walsh (2011). The two dimensions of tourist citizenship behavior (i.e., organizational-directed citizen behaviors and customer-directed citizen behaviors) were measured. A measure of tourism destination brand experience was taken from Brakus et al. (2009). A total of four items about sensory experience, affective experience, behavioral experience, and intellectual experience were measured using a 7-point Likert-type scale. Kim et al. (2014) also employed a measure of brand relationship quality. Five items for self-connection, partner quality of the brand, love/passion, trust, and intimacy were measured using a 7-point Likert-type scale. The two items of commitments, commitment to provider and commitment to fellow people, were measured on a 7-point Likert-type scale from Curth et al. (2014).

### 3.3. Data analysis

#### 3.3.1. Reliability analysis

It can be seen from the reliability test of the research variables in Table 2 that the Cronbach’s value of each variable is greater than 0.7, indicating that the reliability of each variable is good. Therefore, we can say that the measurement indicators of the research variables have high internal consistency and the investigation of the data is relatively reliable.

**Table 2:** Reliability analysis.

Scale	Cronbach’s Alpha	Items
Organizational-directed citizen behaviors	0.893	3
Customer-directed citizen behaviors	0.883	3
Sensory experience	0.896	3
Affective experience	0.883	3
Behavioral experience	0.893	3
Intellectual experience	0.849	3
Self-connection	0.869	3
Partner quality	0.871	3
Love/passion	0.886	3
Trust	0.866	3
Intimacy	0.903	3
Commitment to provider	0.925	7
Commitment to fellow people	0.916	7

#### 3.3.2. Confirmatory factor analysis

This study used Amos 21.0 software to conduct confirmatory factor analysis using the maximum likelihood method to verify the construct validity of the model and scale. When using confirmatory factor analysis to evaluate model fit, it is necessary to consider multiple indicators such as absolute fit, value-added fit, and parsimony fit.

When analyzing the chi-squared degrees of freedom ratio, that is, CMIN/DF in the AMOS output result, the model fit is good when the value is less than 3 means and poor when it is greater than 3 means. Then analyzed, the SRMR value (root mean square residual) is equal to the square root of the mean value of the covariance of the fitting residual equation. The smaller the RMR value, the better the fitting degree of the model. The RMSEA value (root mean square error of approximation) is an absolute indicator that does not require a baseline model. The smaller the value, the better the fit of the model. Generally speaking, a value between 0.08 and 0.10 indicates that the model is acceptable and has normal adaptation; a value between 0.05 and 0.08 indicates that the model has a good degree of fitness and a reasonable degree of adaptation; a value less than 0.05 indicates that the model has a good degree of fitness very good. GFI (goodness-of-fit index) is an absolute index. The closer the value is to 1, the better the fit of the model. IFI (incremental fit index) and CFI (comparative fit index) are relative indicators; they are value-added fitness statistics that usually compare the fitness of the theoretical model to be tested with the baseline model to judge the fitness of the model. The closer the value to 1, the more fit the model is. PNFI (parsimony-adjusted NFI) is the adjustment index of the parsimony fit index, and the ideal value should be above 0.50.

According to the index relationship, we constructed the confirmatory factor analysis model of this study using the data obtained from the questionnaire to carry out the confirmatory factor analysis and optimize and correct the error terms.

Our study shows in Tables 3–5 that the questionnaire model comprises 11 first-order factors on destination brand experience, tourist citizenship behavior and brand relationship quality. The standardized factor loading values of each measurement item was greater than 0.5, the critical ratio C.R. was greater than 1.96, and all were significant at the 0.001 level. In addition, the combined reliability of each factor was greater than 0.7, indicating that the combined reliability of the model is good. The AVE value of the average variance extraction of each factor was greater than 0.5, and the model has good convergent validity.

**Table 3:** Confirmatory factor analysis model.

Goodness-of-fit index	Ideal standard	General standard	Model result	Result
CMIN/DF	1–3	The smaller the better	1.677	Good
RMSEA	<0.08	<0.1	0.046	Good
RMR	<0.08	<0.1	0.07	Good
GFI	>0.90	>0.8	0.875	Fair
CFI	>0.90	>0.8	0.956	Good
IFI	>0.90	>0.8	0.956	Good
PNFI	>0.50		0.797	Good

**Table 4:** Factor structural validity model.

Latent variable	Variables	Factor load	C.R.	*p*	Composite reliability	AVE
Sensory experience	Q2_1	0.841			0.897	0.744
	Q2_2	0.88	18.949	***		
	Q2_3	0.866	18.637	***		
Affective experience	Q2_4	0.844			0.884	0.718
	Q2_5	0.851	17.492	***		
	Q2_6	0.847	17.409	***		
Behavioral experience	Q2_7	0.831			0.895	0.741
	Q2_8	0.915	19.267	***		
	Q2_9	0.833	17.605	***		
Intellectual experience	Q2_10	0.78			0.851	0.656
	Q2_11	0.861	14.657	***		
	Q2_12	0.786	13.964	***		
Organizational-directed citizen behaviors	Q1_1	0.85			0.894	0.738
	Q1_2	0.895	19.491	***		
	Q1_3	0.831	17.898	***		
Customer-directed citizen behaviors	Q1_4	0.832			0.886	0.721
	Q1_5	0.839	17.198	***		
	Q1_6	0.875	17.928	***		
Self-connection	Q3_1	0.826			0.870	0.690
	Q3_2	0.847	16.575	***		
	Q3_3	0.819	16.05	***		
Partner quality	Q3_4	0.79			0.875	0.701
	Q3_5	0.866	16.657	***		
	Q3_6	0.853	16.417	***		
Love/passion	Q3_7	0.822			0.890	0.729
	Q3_8	0.873	17.911	***		
	Q3_9	0.865	17.748	***		
Trust	Q3_10	0.901			0.871	0.693
	Q3_11	0.774	16.55	***		
	Q3_12	0.817	17.86	***		
Intimacy	Q3_13	0.929			0.905	0.760
	Q3_14	0.837	20.62	***		
	Q3_15	0.846	21.028	***		

***show a significant level.

**Table 5:** Discriminant validity test.

$\sqrt[{2}]{AVE}$	Sensory experience	Affective experience	Behavioral experience	Intellectual experience	Organizational-directed citizen behaviors	Customer-directed citizen behaviors	Brand relationship quality
Sensory experience	0.863						
Affective experience	0.367	0.847					
Behavioral experience	0.419	0.249	0.861				
Intellectual experience	0.358	0.174	0.314	0.81			
Organizational-directed citizen behaviors	0.498	0.389	0.42	0.361	0.859		
Customer-directed citizen behaviors	0.411	0.422	0.388	0.366	0.446	0.849	
Brand relationship quality	0.426	0.32	0.442	0.338	0.402	0.402	0.744

The diagonal line is known as the positive square root of the average variance extraction AVE of the corresponding latent variable, and the correlation coefficient between the latent variables is below the diagonal line.

In the fitting index results of the factor model, the Chi-square degree of freedom ratio is 1.667 < 3.000, which indicates that the model has a good degree of fit. In terms of other fitness indicators, the performance of each indicator is relatively good and the overall model fitting situation is good, which is acceptable.

#### 3.3.3. Variable correlation analysis

The study shows that there is a significant positive correlation between each variable, and further regression model analysis was carried out to study the influence of each variable, as shown in Table 6.

**Table 6:** Variable correlation analysis.

		Organizational-directed citizen behaviors	Customer-directed citizen behaviors	Sensory experience	Affective experience	Behavioral experience	Intellectual experience	Brand relationship quality
Organizational-directed citizen behaviors	Pearson’s correlation	1	0.355^**^	0.438^**^	0.348^**^	0.336^**^	0.323^**^	0.391^**^
	Significant (2-tailed)		0.000	0.000	0.000	0.000	0.000	0.000
	*N*	325	325	325	325	325	325	325
Customer-directed citizen behaviors	Pearson’s correlation	0.355^**^	1	0.384^**^	0.374^**^	0.312^**^	0.324^**^	0.386^**^
	Significant (2-tailed)	0.000		0.000	0.000	0.000	0.000	0.000
	*N*	325	325	325	325	325	325	325
Sensory experience	Pearson’s correlation	0.438^**^	0.384^**^	1	0.347^**^	0.294^**^	0.306^**^	0.368^**^
	Significant (2-tailed)	0.000	0.000		0.000	0.000	0.000	0.000
	*N*	325	325	325	325	325	325	325
Affective experience	Pearson’s correlation	0.348^**^	0.374^**^	0.347^**^	1	0.191^**^	0.156^**^	0.275^**^
	Significant (2-tailed)	0.000	0.000	0.000		0.001	0.005	0.000
	*N*	325	325	325	325	325	325	325
Behavioral experience	Pearson’s correlation	0.336^**^	0.312^**^	0.294^**^	0.191^**^	1	0.216^**^	0.299^**^
	Significant (2-tailed)	0.000	0.000	0.000	0.001		0.000	0.000
	*N*	325	325	325	325	325	325	325
Intellectual experience	Pearson’s correlation	0.323^**^	0.324^**^	0.306^**^	0.156^**^	0.216^**^	1	0.296^**^
	Significant (2-tailed)	0.000	0.000	0.000	0.005	0.000		0.000
	*N*	325	325	325	325	325	325	325
Brand relationship quality	Pearson’s correlation	0.391^**^	0.386^**^	0.368^**^	0.275^**^	0.299^**^	0.296^**^	1
	Significant (2-tailed)	0.000	0.000	0.000	0.000	0.000	0.000	
	*N*	325	325	325	325	325	325	325

^**^Significantly correlated at the 0.01 level (two-tailed).

Structural equation modeling usually includes two basic models: the measurement model and structural model. The measurement model is composed of latent variables and observed variables and reflects the relationship between these two types of variables. In contrast, the structural model represents the relationship between latent variables. This is an empirical analysis model method. Structural relationships verify whether the assumptions of a structural relationship or model are reasonable and whether the model is correct. The structural equation model has the advantage of considering and processing multiple dependent variables simultaneously, allowing both independent and dependent variables to contain measurement errors.

**Figure 2:**
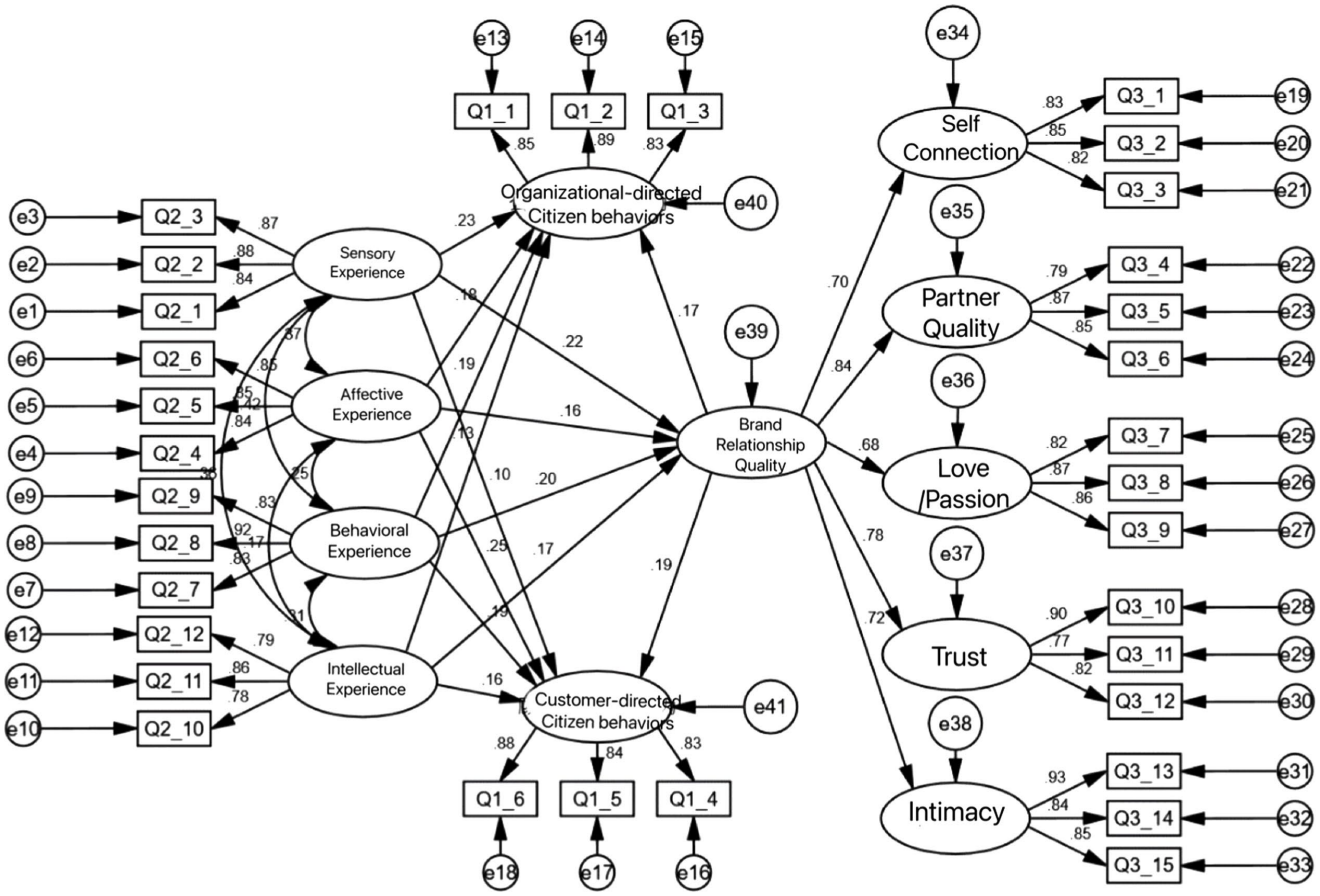
Structural equation path model.

Based on the research theory and assumptions in this study, the influence relationship model between variables was constructed. The path analysis of the influence structural equation between each variable is shown in Figure 2.

In this study, Amos 24.0 software was used to perform structural equation model operations on the collected data. The operating results were sorted and analyzed.

#### 3.3.4. Mediator variable analysis

We tested the mediation variable effect using the bootstrap method. The sample size was 5,000, and the confidence interval was 95%.

The results of the mediation variable test are shown in Table 7. The mediating effect of the tourism destination brand experience on brand relationship and tourist citizenship behavior is 0.032, and the confidence interval is (0.006, 0.081), including 0. This indicates that the mediating effect is significant.

**Table 7:** Mediator variable analysis.

Mediator path	Mediator level	SE	Bootstrap CI (95%)		*p*	Hypothesis
			Lower	Upper		
Intellectual experience-Brand relationship quality-Customer directed citizen behaviors	0.032	0.022	0.006	0.081	0.017	Support
Behavioral experience-Brand relationship quality-Customer directed citizen behaviors	0.039	0.016	0.008	0.096	0.011	Support
Affective experience-Brand relationship quality-Customer directed citizen behaviors	0.03	0.02	0.006	0.074	0.011	Support
Sensory experience-Brand relationship quality-Customer directed citizen behaviors	0.043	0	0.012	0.096	0.006	Support
Intellectual experience-Brand relationship quality-Organizational directed citizen behaviors	0.029	0.017	0.005	0.029	0.014	Support
Behavioral experience-Brand relationship quality-Organizational directed citizen behaviors	0.035	0.019	0.007	0.035	0.007	Support
Affective experience-Brand relationship quality-Organizational directed citizen behaviors	0.027	0.015	0.006	0.027	0.009	Support
Sensory experience-Brand relationship quality-Organizational directed citizen behaviors	0.039	0.02	0.009	0.039	0.004	Support

#### 3.3.5. Moderator variable analysis

##### 3.3.5.1. Factor of commitment to provider analysis

This study considers the brand relationship quality as the independent variable and the organizational-directed citizenship behaviors as the dependent variable to study the influence of the brand relationship quality on the organizational-directed citizenship behavior and study the relationship between the mediating effect of the moderator variable on the commitment to the provider (Table 8).

**Table 8:** Model analysis.

Model	*R*	*R*^2^	Adjustment *R*^2^	Error in standard
1	.458^a^	0.210	0.202	1.12384

^a^means adjust R.

The regression analysis results in Table 9 show that the analysis of variance results show that value F is 28.397 and the corresponding significance probability is 0.000, which is less than 0.05 and corresponds with the significance level. The model fitting effect is good, and the results are remarkably effective.

**Table 9:** ANOVA^a^ analysis.

Model		Sum of squares	Df	Mean square	*F*	Significant
1	Between groups	107.598	3	35.866	28.397	.000
	Within groups	405.429	321	1.263		
	Total	513.027	324			

^a^means adjust ANOVA analysis.

The standard regression coefficient of the interaction item on commitment in Table 10 to provider*brand relationship quality is 0.139. The significance sig value is 0.006 and less than 0.05, which corresponds with the significant level and indicates that the significant level and indicates that the significant positive relationship of the interaction item on commitment to provider*brand relationship quality has a significant effect on the dependent variable, organizational-directed citizenship behavior. Therefore, it has a positive moderating effect on commitment to provider (Table 11).

**Table 10:** Coefficient^a^ analysis.

Model		Unstd. Error in Std.		Std.	*t*	Significant
1	(Constant)	0.759	0.327		2.322	0.021
	Brand relationship quality	0.490	0.086	0.302	5.701	0.000
	Commitment to provider	0.271	0.073	0.195	3.708	0.000
	Commitment to provider * Brand relationship quality	0.240	0.088	0.139	2.741	0.006

^a^Dependent Variable: Organizational-directed Citizenship Behaviors.

**Table 11:** Model analysis.

Model	*R*	*R*^2^	Adjustment *R*^2^	Error in standard
1	.411^a^	0.169	0.161	0.93029

^a^means adjust R.

##### 3.3.5.2. Analysis of factors of commitment to fellow people

The regression analysis results show in Table 12 that the value *F* is 21.714 and the corresponding significance probability is 0.000, which is less than 0.05 and corresponds with the significance level. The model fitting effect is good, and the results are remarkably effective.

**Table 12:** ANOVA^a^ analysis.

Model		Sum of squares	Df	Mean square	*F*	Significant
1	Between groups	56.377	3	18.792	21.714	.000
	Within groups	277.806	321	0.865		
	Total	334.183	324			

^a^means adjust ANOVA analysis.

In Table 13, the standard regression coefficient of the interaction item on the commitment to fellow people*brand relationship quality is 0.128. The significant sig value is 0.015 and less than 0.05, which corresponds to a significant level and indicates that the interaction item has a significant relationship with the commitment to fellow people*brand relationship quality on the dependent variable, organizational-directed citizenship behavior. Therefore, it has a positive moderating effect on the commitment to fellow people.

**Table 13:** Coefficient^a^ analysis.

Model		Unstd. error in Std.		Std.	*t*	Significant
1	(Constant)	1.650	0.286		5.761	0.000
	Brand relationship quality	0.463	0.069	0.354	6.717	0.000
	Commitment to fellow people	0.054	0.062	0.046	0.869	0.386
	Commitment to fellow people * Brand relationship quality	0.194	0.079	0.128	2.453	0.015

^a^Dependent Variable: Customer-directed Citizenship Behaviors.

## 4. Conclusion and implications

Many researchers have investigated the impact of brand experience on citizenship attitudes (Gao et al., 2016). Previous research has often overlooked explanations for why and how different brand experiences positively encourage citizen behaviors. Therefore, this study attempts to expand the understanding of the impact of tourism destination brand experience on tourist citizenship behavior. The conceptual framework was developed based on the S-O-R framework.

This study contributes to the existing tourism literature in four ways. First, the proposed model was tested by integrating the S-O-R framework to describe the stages involved in tourism destination brand experience, brand relationship quality, and tourist citizenship behavior in the tourism industry. Second, the study highlights the important mediator role of brand relationship quality in explaining why and how tourism destination brand experience changes citizen behaviors in the tourism industry. Third, the study demonstrates the moderator role of commitment to analyze the relationship between brand relationship quality and tourist citizenship behavior. Finally, this study applied an integrative framework, including stimulus, organismal, and mental response models to improve the understanding of tourist citizenship behavior.

Therefore, this study seeks to improve the understanding of how brand relationship quality acts as an influencer (mediator) and commitment plays a moderating role on the relationship between tourism destination brand experience and tourist citizenship behavior by integrating the S-O-R framework to explain tourism in Guangzhou city.

## 5. Theoretical contributions

Based on the integration of the S-O-R framework, the model treats the concepts of tourism destination brand experience, brand relationship quality, and tourist citizenship behavior as the stimulus, organism, and response, respectively. Previous research has demonstrated the impact of brand experience on customers’ attitudes in the tourism industry; however, limited research has been conducted on how these experiential factors translate into behavioral factors (Gao et al., 2016). We found that brand relationship quality has a significant mediating role in the stages involved in tourism destination brand experience and tourist citizenship behavior. In addition, we highlighted that commitment plays an important significant moderating role on brand relationship quality and tourist citizenship behavior.

The most important contribution of this study is that this study analyses the S-O-R framework and analyses the mediating role of brand relationship quality between tourism destination brand experience and tourist citizenship behavior as well as the moderating role of commitment between brand relationship quality and tourist citizenship behavior. This study treats behavioral consequences (e.g., tourist citizenship behavior) as a response to the S-O-R framework because, according to the S-O-R framework, brand relationship quality includes self-connection, partner quality, love/passion, trust, and intimacy as an integral part of an organism. Therefore, this study contributes to the literature by explaining the underlying mechanisms of tourist citizenship behavior in the tourism industry. Considering the increasing number of tourists visiting Guangzhou city, the proposed model can be applied to examine the impact of tourism destination brand experience and tourist citizenship behavior in Guangzhou city.

Furthermore, the proposed framework contributes to the existing tourism literature by considering the mediating role of brand relationship quality between tourism destination brand experience and tourist citizenship behavior. We found that in the tourism industry, tourism destination brand experience has a significant impact on tourist citizenship behavior. This study extends the findings of Gouthier and Schmid (2003) by determining that citizen behavior appears to be the most effective way to increase service productivity. We found that brand relationship quality significantly influences tourist citizenship behavior. We also found that brand relationship quality is the result of the tourism industry’s destination brand experience. Therefore, in the tourism industry, it is very important to understand brand relationship quality, as it is an important intermediary in translating tourism destination brand experience into tourist citizenship behavior. As expected, commitment also significantly influenced brand relationship quality, and tourist citizenship behavior played a moderating role. This result explains how tourist citizenship behavior is triggered. Therefore, this study improves the understanding of researchers in the tourism industry on brand relationship quality to encourage tourist citizenship behavior.

These findings suggest that tourism destination brand experience has significant direct effects on tourist citizenship behavior. In other words, tourists who visited Guangzhou city tended to be highly inspired when Guangzhou city offered sensory, emotional, behavioral, and intellectual experiences. The brand relationship quality of Guangzhou city may increase when they indulge in sensory, emotional, behavioral, and intellectual experiences. The results show that tourism destination brand experience is related to tourist citizenship behavior when visiting Guangzhou city. By following previous marketing literature (Böttger et al., 2017), the results of the current study extend the understanding of tourist citizenship behavior in the tourism and hospital industries.

Overall, we found that brand relationship quality and commitment can be new incentive variables that researchers and service providers should focus on, as incentives influenced by tourism destination brand experience have a significant positive effect on tourist citizenship behavior. In addition, commitment plays an important role in increasing individuals’ tourist citizenship behavior. Tourists with good commitment tend to have better experiences and are more satisfied when visiting Guangzhou city, as brand relationship quality positively correlates with tourist citizenship behavior.

## 6. Practical contributions

The findings suggest that sensory, emotional, behavioral, and intellectual experiences from tourism destination brands inspire citizens. The perceptions of citizens who experience pleasant sensory cues, positive emotions, behavioral activities, or interactions with tourism staff during their visit to Guangzhou city are enhanced, which in turn enhances their tourist citizenship behavior. Therefore, the advantages of developing memorable and enjoyable experiences and increasing tourist citizenship behavior also apply to visiting Guangzhou city in a sustainable tourism industry.

Tourism marketers need to use a variety of experiential factors, such as sensory, emotional, behavioral, and intellectual, to identify what contributes to citizens’ experiences. Guangzhou city should provide attractive sensory cues to increase brand relationship quality. Additionally, Guangzhou city may suggest technological devices in order to provide tourists with a unique behavioral or intellectual experience while visiting Guangzhou city and to heighten the quality of brand relationship quality. For example, some historical sites in Guangzhou city can use robotic docents, allowing individuals tourist to actively interact with the robot, thereby increasing the level of brand relationship quality in Guangzhou for tourists. Therefore, service providers need to focus on designing attractive sensory cues, creating positive feelings, improving pleasant service, and training staff to attract tourists, as these experiences are associated with increasing levels of brand relationship quality. Considering the impact of tourism destination brand experience on brand relationship quality, companies should develop tourism destination brand experience through a variety of experiences that evoke brand relationship quality.

The findings support a strong positive relationship between brand relationship quality and tourist citizenship behavior. Increasing Guangzhou city’s brand relationship quality for the tourism destination brand experience is the key to successfully encouraging tourists to visit Guangzhou city, as it increases tourists’ willingness to visit Guangzhou city. The research results also prove the important moderating role of commitment, including that of the commitment to provider and commitment to fellow people in Guangzhou city. Tourists who experience sensory, emotional, behavioral, and intellectual experiences tend to have a good relationship with and travel more to Guangzhou city. As a result, Guangzhou city can develop tourism destination brand experiences to increase its citizens’ level of brand relationship quality and commitment to the city, which ultimately increases tourist citizenship behavior.

## 7. Limitations and future research directions

While this study contributes to existing literature on sustainable tourism, it has limitations. First, the samples were collected in China. Future research could replicate the proposed model in different samples in the United States and Europe to determine whether tourist citizenship behavior varies by culture (Kang et al., 2017). Second, this study was conducted through an online survey. Future research could include interviews with tourists to identify influencing factors that may influence brand relationship quality and tourist citizenship behavior. Various approaches may help understand tourist citizenship behavior better. Third, factors, including tourism destination brand experience, brand relationship quality, and tourist citizenship behavior, were measured based on the questionnaire provided by the authors. To determine actual behavior, future researchers can employ a hybrid approach of field and experimental studies to investigate the actual impact of tourists’ behavior.

## Data availability statement

The original contributions presented in the study are included in the article/supplementary material, further inquiries can be directed to the corresponding authors.

## Author contributions

JT and MZ designed the work and were responsible for the overall development of this article. JT was in charge of data collection and analysis of this study and the main revision for this manuscript and also made a great contribution to the final acceptance of the manuscript. MZ was planning sample collection, data analysis, writing, and polishing of the manuscript. All authors contributed to the article and approved the submitted version.

## Funding

This research is funded by: A Study on Guangzhou’s Development of a High-quality Tourism Industry under the background of Cultural and Tourism Integration, Guangdong Federation of Social Sciences (2021GZGJ53); Research on the High-quality Development of Heyuan Service Industry under the New Development Pattern of “double circulation”, Heyuan Federation of Social Sciences (HYSK21P68).

## Conflict of interest

The authors declare that the research was conducted in the absence of any commercial or financial relationships that could be construed as a potential conflict of interest.

## Publisher’s note

All claims expressed in this article are solely those of the authors and do not necessarily represent those of their affiliated organizations, or those of the publisher, the editors and the reviewers. Any product that may be evaluated in this article, or claim that may be made by its manufacturer, is not guaranteed or endorsed by the publisher.
